# Sister Mary Joseph Syndrome: A Report of a Rare Case

**DOI:** 10.7759/cureus.64707

**Published:** 2024-07-17

**Authors:** Imane Bellahyane, Mohamed Moukhlissi, Meriem Bouabid, Soufiane Berhili, Loubna Mezouar

**Affiliations:** 1 Radiation Oncology, Faculty of Medicine and Pharmacy, Mohammed First University Mohammed, Oujda, MAR

**Keywords:** endometrial endometrioid adenocarcinoma, abdominopelvic cancer, umbilical nodule, cutaneous metastases, sister mary-joseph

## Abstract

The Sister Mary Joseph syndrome is characterized by cutaneous metastases localized at the umbilical level. It is a rare clinical sign estimated to occur in 1%-3% of patients with abdominopelvic cancer. The most common histology is adenocarcinoma (75% of cases). The presence of this nodule is often indicative of a poor prognosis, with average survival estimated at two to 11 months without treatment. We report the clinical case of Sister Mary Joseph syndrome in a 50-year-old woman who had been followed for three years for endometrioid adenocarcinoma of the endometrium. The diagnosis was established via umbilical biopsy after a computed tomography scan revealed the presence of an umbilical nodule. The patient is currently undergoing a palliative chemotherapy regimen.

## Introduction

Sister Mary Joseph syndrome, first described in 1928 by Mary Joseph, established the relationship between the presence of an umbilical nodule and intra-abdominal cancers [1,2]. Usually, urogenital or digestive tumors are the original malignancies [3]. The nodule is often associated with an advanced malignant tumor with a poor prognosis [4]. We present the case of a patient who had endometrioid cancer of the endometrium and developed an umbilical nodule as her illness progressed.

## Case presentation

A 50-year-old patient, followed for three years for endometrioid adenocarcinoma of the endometrium, was discovered incidentally after a subtotal hysterectomy performed for hemostatic purposes due to a large polyp visualized on pelvic ultrasound. An anatomopathological study revealed the presence of grade II endometrioid carcinoma of the endometrium, with infiltration of more than 50% of the myometrium; vascular emboli were negative. Immunohistochemistry showed positive hormone receptors, with 40% for estrogen and 60% for progesterone. Revision surgery was performed to complete the hysterectomy and conduct adnexectomy and lymph node dissection, which revealed five metastatic lymph nodes. A postoperative computed tomography (CT) scan revealed a secondary pulmonary location.

The patient received six cycles of palliative chemotherapy consisting of carboplatin (area under the curve [AUC] 6) and paclitaxel (175 mg/m²) every three weeks, resulting in a good response and complete remission of secondary lung lesions, as assessed by CT scan. Subsequently, concurrent radiochemotherapy was administered, and hormone therapy was initiated for the patient.

**Figure 1:**
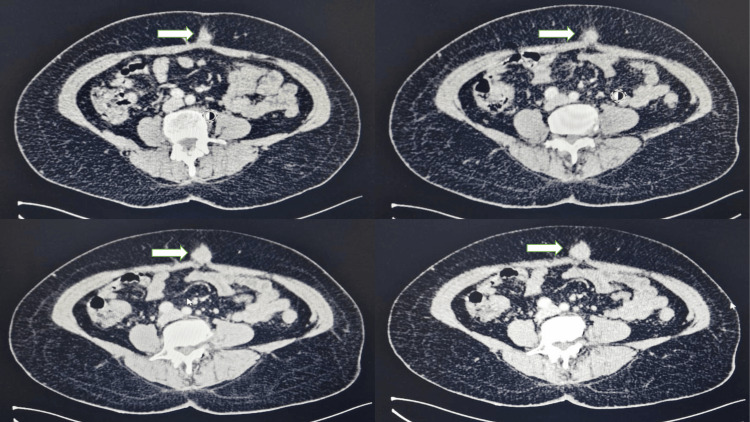
A computed tomography scan revealed the presence of an umbilical nodular formation, oval-shaped, hypodense, and enhanced after contrast injection (arrow), suggestive of a Sister Mary Joseph nodule.

The patient remained well-controlled. Two years later, a follow-up CT scan revealed the presence of an umbilical nodule, measuring 22 mm x 21 mm, suggestive of a Sister Mary Joseph nodule (Figure. 1).

**Figure 2:**
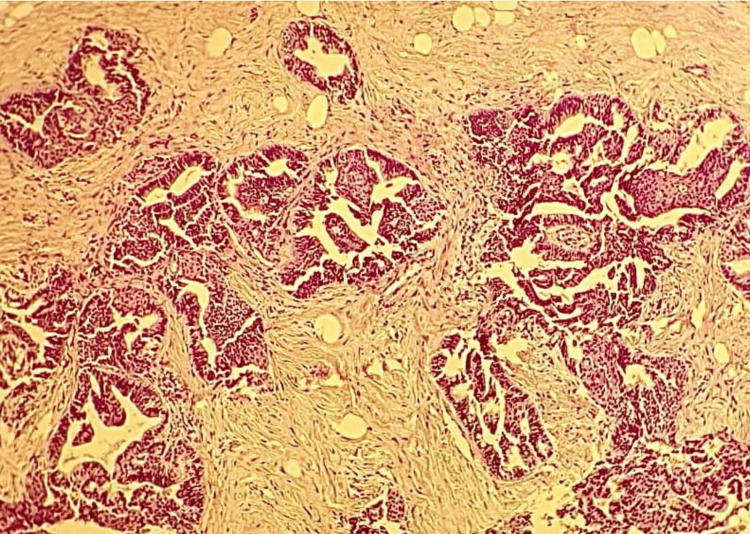
Histological image showing fibrous tissue with carcinomatous proliferation arranged in glands and cribriform masses (HES, x20). HES, Hematoxylin-Eosin-Saffron

Additionally, secondary lesions were observed in the lung and peritoneum. A biopsy of the umbilical lesion was performed, and histopathological examination showed fibrous tissue extensively infiltrated by a carcinomatous proliferation composed of glands and cribriform masses, suggestive of a secondary localization of endometrioid adenocarcinoma of the endometrium (Figure 2).

The patient is currently undergoing the same palliative chemotherapy regimen.

## Discussion

The Sister Mary Joseph syndrome is characterized by cutaneous metastases localized at the umbilical level, typically presenting as a palpable umbilical skin nodule [5]. Sister Mary Joseph (1856-1939) was the first to observe the presence of a firm umbilical nodule in patients operated on for abdominal malignant tumors. However, Hamilton Bailey first described it in his book *Physical Signs in Clinical Surgery* [5,6]. According to epidemiological data, umbilical metastases are rare and estimated to occur in 1%-3% of patients with abdominopelvic cancer [7]. The male-to-female sex ratio is 0.69, indicating a predominance of females [7].

There is still much to learn about the process underlying tumor propagation to the umbilicus. Several hypotheses have been proposed. It primarily concerns hematogenous dissemination due to the rich arterial vascularization of the umbilicus, as well as lymphatic dissemination. The umbilical region is drained by a lymphatic system consisting of a deep network (involving para-aortic, internal mammary, and external iliac lymph nodes) and a superficial network (involving axillary and inguinal lymph nodes). Lymphatic dissemination is the more important mechanism of spread, exerting a major impact on prognosis and surgical management [8]. Other hypotheses explaining the presence of tumor cells in this umbilical region include direct invasion of the peritoneum or accidental implantation following surgical intervention [8,9].

Adenocarcinoma is the most frequent primary histological type, accounting for 75% of cases, which is also found in our patients. However, in rare instances, it may also manifest as squamous cell carcinoma or undifferentiated carcinoma [8]. In men, digestive cancers are predominant, with gastrointestinal cancer accounting for 30% of instances, colorectal cancer for 25%, and pancreatic cancer for 18%. However, in women, gynecological cancer is the most common cause of cancer, with ovarian cancer accounting for 34% of cases, endometrial cancer for 12%, and cervical cancer for 5%. In our case, it was an endometrioid adenocarcinoma of the endometrium [3,6].

Umbilical metastases typically present as irregular, firm, or elastic nodules of varying sizes, which may also be painful, ulcerated, or oozing. Clinically, these lesions are often indistinguishable from other benign lesions. In our case, the mass appeared deeper and was not clinically apparent [7,8].

Radiological examinations such as ultrasonography, CT, magnetic resonance imaging, and positron emission tomography are necessary and can orient the diagnosis; however, they are often insufficient. Once Sister Marie Joseph syndrome is discovered on imaging, the anatomopathological study is essential to confirm the diagnosis and identify the primary tumor, thereby eliminating differential diagnoses [2,4].

Differential diagnoses include umbilical endometriosis, umbilical hernias, benign umbilical primary tumors (epidermal cysts, nevi, keloids, etc.), or malignant tumors such as basal or squamous cell carcinomas, melanomas, sarcomas, etc., or umbilical localizations of Crohn's disease, pyogenic or foreign-body granulomas, or hemangiomas [7,10].

Treatment is typically palliative. However, intervention surgical combined with chemotherapy can improve survival, and patients treated with surgery followed by adjuvant therapy have a higher average survival than those treated with chemotherapy alone or surgery alone [4,11].

The presence of this nodule is often indicative of a poor prognosis, as it usually presents an advanced, disseminated stage of the tumor. Average survival is estimated at two to 11 months without treatment [12].

## Conclusions

Sister Mary Joseph syndrome is an uncommon but distinctive symptom. It emphasizes how crucial it is to perform a comprehensive clinical examination of the abdomen, especially in patients who have intra-abdominal cancers, to identify any umbilical lesion. The most reliable method for diagnosing any suspicious lesion emerging from the umbilicus is through a biopsy.
